# *Galleria mellonella*—A Model for the Study of aPDT—Prospects and Drawbacks

**DOI:** 10.3390/microorganisms11061455

**Published:** 2023-05-31

**Authors:** Larysa Bugyna, Samuel Kendra, Helena Bujdáková

**Affiliations:** Faculty of Natural Sciences, Department of Microbiology and Virology, Comenius University in Bratislava, Ilkovicova 6, 84215 Bratislava, Slovakia; larysa.bugyna@uniba.sk (L.B.); kendra4@uniba.sk (S.K.)

**Keywords:** *Galleria mellonella*, larvae infection, antimicrobial photodynamic therapy, photosensitizer, irradiation

## Abstract

*Galleria mellonella* is a promising *in vivo* model insect used for microbiological, medical, and pharmacological research. It provides a platform for testing the biocompatibility of various compounds and the kinetics of survival after an infection followed by subsequent treatment, and for the evaluation of various parameters during treatment, including the host–pathogen interaction. There are some similarities in the development of pathologies with mammals. However, a limitation is the lack of adaptive immune response. Antimicrobial photodynamic therapy (aPDT) is an alternative approach for combating microbial infections, including biofilm-associated ones. aPDT is effective against Gram-positive and Gram-negative bacteria, viruses, fungi, and parasites, regardless of whether they are resistant to conventional treatment. The main idea of this comprehensive review was to collect information on the use of *G. mellonella* in aPDT. It provides a collection of references published in the last 10 years from this area of research, complemented by some practical experiences of the authors of this review. Additionally, the review summarizes in brief information on the *G. mellonella* model, its advantages and methods used in the processing of material from these larvae, as well as basic knowledge of the principles of aPDT.

## 1. Introduction

In modern medicine and pharmaceutical research, the selection of the appropriate choice of *in vivo* model has been critical [1,2,3,4,5,6]. Research involving vertebrate animals is subject to strict rules and introduces a number of ethical problems. The European Science Foundation promotes the need for an ethical approach to each animal experiment. In 1986, the Council of Europe and the European Union (EU) issued guidelines and legislation on the use of animals for scientific purposes. Several organizations have prepared guidelines for their ethical use, and in many countries this is controlled at the level of national legislative norms. For EU members, national legislation must meet the requirements of Council Directive 2010/63/EU of the European Parliament, which has been updated from time to time [7,8]. Planning any research that involves animals requires following the rules of the “3 Rs”—replace, reduce, refine. This means that firstly, if possible, it is necessary to replace vertebrates with invertebrates; secondly, if this is impossible, it is important to reduce their use to a minimum; and thirdly, to refine the research in such a way as to minimize the suffering of vertebrates. At the same time, obtaining reliable results should be ensured [8,9]. Such standards are not so strict for invertebrates, such as the nematode *Caenorhabditis elegans* [10,11], the fruit fly *Drosophila melanogaster* [12,13], zebrafish *Danio rerio* [14,15], and the wax moth larvae of *Galleria mellonella*. The latter is a universal invertebrate model suitable for conducting various studies that evaluate many different parameters [4,16,17,18,19,20,21,22].

*G. mellonella* has several advantages over other non-vertebrate models (mentioned in detail in the next section). Additionally, it has some similarities to mammals in terms of the development of pathologies with mammals [2,4,23]. *G. mellonella* larvae have a wide applicability for each type of assay, which may inform the prospects for further *in vivo* studies in mammals. However, their limitation is a lack of adaptive immune response, namely the elevation of antibodies and cytokines and the participation of leukocyte killer cells and dendritic cells, which makes it difficult to predict the immune response in mammals [23]. Another obstacle is the lack of availability of mutant larvae, which makes genetic studies problematic [2]. On the other hand, the inoculation of *G. mellonella* larvae is rapid, so results can be obtained within a few days. This model is suitable not only for testing the biocompatibility of different compounds and the kinetics of survival after an infection followed by subsequent treatment, but also for the evaluation of different parameters during treatment, including the host–pathogen interaction [4,16,17,24,25].

Antimicrobial photodynamic inactivation (aPDI) is an alternative strategy for fighting against microorganisms and their biofilms. This approach is based on the use of a non-toxic dye-photosensitizer (PS), a source of light with the proper wavelength, and the presence of oxygen. The optimal interplay of all the above factors results in oxidative stress leading to the death of target cells [26,27]. In aPDI, there is no specifically targeted component, but it causes general damage in the cell. Therefore, it is more difficult for microorganisms to adapt, or even to develop resistance [28].

The main idea of this comprehensive review was to collect information on the use of *G. mellonella* in the study of antimicrobial photodynamic therapy (aPDT). Moreover, the review summarizes in brief information on the *G. mellonella* model, its advantages, and the methods used in the processing of material from these larvae. The information from published works is complemented by the experimental experiences of the authors of this review.

## 2. General Characterization of *G. mellonela* and Significance for Microbiological Research

The larvae of *G. mellonella* inhabit honeybee hives and feed on bee honeycombs, where their subsequent pupation takes place. The duration of the life cycle is 8–12 weeks, including 5–6 weeks in the larval stage, and larvae are usually about 3 cm long [29,30,31,32,33,34]. The advantages of *G. mellonella* compared to other invertebrate experimental objects can be summarized as follows: (a) larger larval dimension, which facilitates experimental manipulations; (b) ability to actively grow over a wider temperature range (20 °C–37 °C); (c) shorter period of data acquisition (several days compared to the weeks of other invertebrate experimental objects). In addition, the data acquired on *G. mellonella* are comparable to the studies obtained on vertebrate animals [2,35,36,37] as larvae can be tested at the physiological temperature of vertebrates. This is an important factor that allows the study of temperature-dependent virulence factors [24,29,38,39,40,41,42,43]. In addition, this invertebrate model is capable of reproducing the clinical signs observed in human infections [42,44,45].

Additional opportunities were identified after the *G. mellonella* genome was successfully sequenced. The level of homology between *G. mellonella* and humans, mice, or other model organisms has been determined [46,47]. Lange *et al.* (2018) published the results from genome sequencing using PacBio’s long-read technology. They showed that the *G. mellonella* genome consists of 37 genes coding for 13 proteins, 2 rRNA, and 22 transport RNA. These results greatly contributed to the wider use of this invertebrate model and the replacement of vertebrates in biomedical research [48,49,50,51].

Previously, there were no standardized larvae of *G. mellonella*, which was a significant obstacle to its wider use. For many years, they were only commercially available as food for reptiles and birds, so they were bred, raised, and kept under various conditions. However, they are now bred specifically for research without the addition of antibiotics or hormones to the feed. Their age and weight are also monitored, and the cuticle is disinfected to prevent infections in control groups [3,52]. The most common method of infection of *G. mellonella* larvae is subcutaneous microinjection. Pathogens can also be administered orally, but commercially available larvae are in the final stage of maturation before pupation, during which time they are almost non-feeding. In this regard, oral administration is carried out using a special probe or at an early stage of maturation [52,53,54].

*G. mellonella* is an excellent experimental model for the preliminary screening of the toxicity and antimicrobial activity of various compounds and disinfectants [3,5,55,56,57,58]. For example, manganese-based compounds [Mn(bpqa-κ^3^N)(CO)_3_]Br, [Mn(bqpa κ^3^N)(CO)_3_]Br, [Mn(CO)_3_(tqa-κ^3^N)]Br, and [Mn(CO)_3_(tpa-κ^3^N)Br, which damage the integrity of the bacterial membrane, demonstrated antibacterial properties with no toxicity to *G. mellonella* [59]. Similarly, silver nanoparticles (AgNPs) tested by Thomaz *et al.* (2020) demonstrated effective antimicrobial activity against *Pseudomonas aeruginosa* [60]. Larva intra-hemocoel injections were carried out with the antimicrobial peptide (Naphthalene-2-ly)-acetyl-diphenylalanine-dilysine-OH (NapFFKK-OH) to test its activity against gram-positive and gram-negative microorganisms [61].

Due to the increase in resistance to antibiotics, silver impurities are widely used, from lunch boxes to medical device implants. For example, the effectiveness of silver acetate against the carbapenem-resistant *Acinetobacter baumannii* was investigated. Using this compound, the infection of *G. mellonella* larvae was under control, leading to significantly improved survival. This study also demonstrated the selective toxicity of silver acetate to bacterial pathogens without harmful effects on larvae [62]. In another study, the effectiveness of probiotics was studied. Larvae were pre-inoculated with one of two commonly used probiotic bacteria, *Lactobacillus rhamnosus GG* [63,64] or *Clostridium butyricum Miyairi* 588 [63], and then challenged with *Salmonella enterica Typhimurium*, enteropathogenic *Escherichia coli*, or *Listeria monocytogenes* [44,65,66]. The survival rates were increased in larvae pre-treated with probiotics compared to the control group inoculated with pathogens alone. Hematocyte density also increased, indicating that both probiotics evocated an immune response [63]. It was also established that *G. mellonella* larvae can be used to assess the virulence of anaerobic bacteria of *Clostridium perfringens* [67,68]. The results demonstrated that *C. perfringens* infection activated the melanization pathway, leading to melanin deposition. Another study proved the effectiveness of available antibiotics against the biofilms of multi-drug-resistant *Pseudomonas aeruginosa* and *Klebsiella pneumoniae* strains [69]. In addition, the use of *G. mellonella* larvae makes it possible to evaluate the antibacterial efficiency of various plant extracts and their ability to modulate the immune response. For example, pomegranate glycolic extract was effective against *Porphyromonas gingivalis*, and it prolonged larval survival compared to the untreated control [70].

In summary, *G. mellonella* has been used for testing many infections caused by different gram-positive and gram-negative bacteria. Among gram-positive microorganisms, *Staphylococcus aureus* [71,72,73,74,75,76,77], *Streptococcus pyogenes* [78,79,80], *Streptococcus pneumoniae* [81,82,83], *Streptococcus mutans* [19,20,84,85,86], *L. monocytogenes* [4,44,65,66,87,88], *Enterococus faecalis* [89,90,91,92], *Enterococcus faecium* [93,94,95,96], *L. rhamnosus GG* [63,64], *C. butyricum Miyairi 588* [63], *C. perfringens* [67,68], *Mycobacterium bovis* [23], *Mycobacterium abscessus* [97,98], and *Mycobacterium tuberculosis* [99,100,101,102] were mentioned. Among gram-negative bacteria, *E. coli* [103,104,105,106], *S. enterica Typhimurium* [107,108,109], *K. pneumonia* [110,111,112,113], *A. baumanii* [39,114,115,116,117], *Francisella tularensis* [118,119,120], *P. aeruginosa* [60,121,122], and *P. gingivalis* [70,123,124] have been involved. *G. mellonella* was also used for testing representatives of fungal pathogens, such as *Candida albicans* [21,25,125,126,127,128], *Candida dubliniensis* [21,25,129,130]*, Aspergillus fumigatus* [38,131,132], *Cryptococcus neoformans* [57,133,134,135]*,* and *Madurella mycetomatis* [45,136,137,138], and also viruses [139,140,141,142] and bacteriophages [143,144,145,146,147,148,149,150].

It has already been mentioned that *G. mellonella* larvae are a suitable *in vivo* model for studies related to drug safety and efficacy. Additionally, they can be used for the study of host–pathogen interactions [2,151,152]. An advantage is the very good survival of *G. mellonella* at the temperature of the human body, and that they often exhibit symptoms of the pathogenesis of various diseases similar to those manifested in humans [4,79,97,153,154,155]. For example, larvae infected with streptococci manifested clear signs of invasive infection. Specifically, these included melanization and the formation of a destructive abscess-like lesion at the inoculation site. These abscesses consisted of dense necrotic tissue in the center and microorganisms. They were surrounded by a band of host hemocytes, coagulated hemolymph, and the extracellular pigment melanin. According to the authors, these features are similar to the histopathology commonly seen in mouse and monkey models and could also be compared with severe soft-tissue infections observed in humans [76].

The protection of *G. mellonella* from microbial infection has been under intensive study, and there are some similarities with humans. While the cuticle mimics the skin [151,152,153,154], the immune response mechanism shows signs related to the innate immunity of vertebrates [156,157,158,159]. Hemocoel contains hemocytes with a similar function to human neutrophils that participate in phagocytosis, during which reactive oxygen species (ROS) are generated [160,161,162,163]. The *G. mellonella* larvae enable the study of the influence of infection on the development of oxidative stress and the antioxidant defense system. Additionally, it has been observed that apoptosis can be initiated during infection [164]. Insect hemocyte extracellular traps (IHETs) were recently described. IHETs act via hemolymph coagulation and melanization, which contributes to the immobilization and killing of bacteria. These processes are mediated by a significant release of hemocytes in *G. mellonella* [156].

Soluble effector molecules orchestrate the humoral response and include complement-like proteins, such as melanin, and antimicrobial peptides, such as gallerimycin and galiomicin, which protect *G. mellonella* from fungal infection, or cecropin, which proved to be effective during infection with *Mycobacterium bovis* BCG lux [21,23,46,165,166,167].

Several approaches are currently available, and these have been adapted and optimized to study various processes in *G. mellonella*. This analysis could be summarized into two main areas of study: (i) the kinetics of survival after the testing of an infection/treatment; (ii) the host–pathogen interaction. The latter scope includes quantitative analysis, but also qualitative ones that consider hemolymph analysis. Both areas can also involve some molecular biology approaches. Some procedures optimized for the *G. mellonella* study are briefly mentioned below with the relevant references.

To determine the progress of infection and the effectiveness of treatment, counting the number of dead larvae, progress in melanization, the direct counting of pathogens in body tissues, and histology could be options for evaluation. When determining the host–pathogen interaction, the most frequent approach is a quantitative analysis providing information on the number of hemocytes in the hemolymph, the results of which can give an idea of the level of the immune response [168,169,170,171]. For this purpose, counting the density of hemocytes, recalculated per 1 mL of hemolymph, can also be conducted [172,173]. The viability of hemocytes can be investigated using an MTT colorimetric assay. [174]. To characterize the different types of hemocytes, light or phase-contrast microscopy, Giemsa staining, or neutral red staining is recommended. The enzymatic activity of hemolymph can be determined by measuring the concentration of insect enzymes involved in immunity, such as lysozyme and superoxide dismutase [171,175,176,177]. The label-free quantification and untargeted analysis of the complete protein profile of hemolymph is usually performed by proteomic analysis, or proteins can be identified via 2D electrophoresis [23,175,178,179,180,181,182,183,184].

The implementation of molecular biology is necessary to obtain more detailed information from all the above-mentioned aspects of the study of *G. mellonella*. For the expression of genes coding for antimicrobial peptides and immunity-related genes, a quantitative RT-PCR or transcriptomic analysis is optimal [23,175,178,184,185,186].

The *in vitro* analysis of phagocytosis is performed using fluorescent microscopy of spotted fluorescent bacteria [164,172,174,187,188]. Phagocytosis in hemolymph *in vivo* is analyzed using the same method [189]. A detailed study of macrophage activation is necessary to understand the level of release of ROS or nitrogen species, as well as regulatory enzymes [190]. Macrophage activation is investigated using the Greiss assay, which analyzes the release of active nitrogen forms [37]. More detailed studies of macrophage activation are related to the study of DNA damage (ELISA), lipid peroxidation level (malonic aldehyde level), catalase level (fluorometric resorufin assay), or superoxide dismutase (ELISA) [160,190,191].

In addition to the above-mentioned conventional methods, *G. mellonella* is a suitable model for the study of various aspects of aPDT. This issue is addressed in the following chapter, including a table summarizing the experimental research over approximately the last 10 years, since the first work on testing aPDT on *G. mellonella* was published.

## 3. Principles of aPDT and the Use of *G. mellonella* in aPDT

Photodynamic therapy (PDT) was discovered more than a century ago. Its essence was revealed in detail by Raab, who published a study in 1900 on the use of aPDT as a cytotoxic technique designed to treat tumors, as well as infectious pathologies [192,193].

The therapeutic effect of PDT (aPDT) is achieved using a photosensitizer (PS) that is irradiated with light, the emission spectrum of which corresponds to the absorption spectrum of the PS. In the presence of molecular oxygen, ROS (superoxide, hydroxyl radical, etc.) and singlet oxygen are generated, which cause the irreversible destruction of a large range of biomolecules, including nucleic acids, lipids, and proteins [58,93,194]. PDT is already in use as an alternative approach for the control of malignant diseases. A review by Ferreira dos Santos *et al.* (2019) nicely summarized [179] the current state of the art in PDT research and treatment focused on cancer. The authors also introduced in detail PSs for practical use in the treatment of many cancers. For example, Porfimer, sodium (Photofrin) was the first PS approved by the Canadian Health Agency in 1993 for the treatment of bladder cancer. In 1998, the U.S. Food and Drug Administration approved it for the treatment of early-stage lung cancer. Currently, 11 additional countries in Europe have accepted the practical use of this PS [195]. While the PDT practiced in cancer therapy is developing dramatically, the application of aPDT for the eradication of pathogenic microorganisms and viruses is still at a very early stage and has only been developing faster in the last decade. Nevertheless, several examples of the practical application of aPDT have already been described, mainly in the treatment of oral diseases. A clinical study by Fonseca *et al.* (2022) demonstrated that aPDT reduced the number of infected anatomical sites in patients with oral candidiasis [196]. Another clinical study by Shetty *et al.* (2022) proved that a single session of aPDT as an adjunct to mechanical debridement is effective at reducing peri-implant soft tissue inflammation and oral yeast colonization in patients with peri-implant mucositis [197]. Alves-Silva *et al.* (2023) used aPDT as an adjunct to a chemo-mechanical preparation, and it was effective at improving root canal disinfection and reducing the lipopolysaccharide and lipoteichoic acid levels in teeth with primary endodontic infection [198].

Generally, aPDI should be an effective method for the eradication of a wide range of microorganisms, including both gram-positive and gram-negative bacteria, viruses, fungi, and parasites [26,27,28,199,200,201]. Due to the fact that PDI is multi-targeted, microorganisms are not able to develop resistance [202,203]. Moreover, PDI is highly effective against microorganisms resistant to conventional antimicrobials [204,205,206,207,208]. For instance, Štefánek *et al.* (2022) used aPDI for the eradication of *Candida auris* biofilms resistant to antifungal agents. They found that aPDI significantly decreased the survival of *C. auris* biofilm cells, and thus proved to have great potential for the eradication of multi-resistant yeasts. Furthermore, the observed upregulation of the *MDR1* and *CDR1* genes did not affect the overall efficacy of methylene blue-mediated aPDI on biofilms formed by *C. auris* clinical isolates, regardless of their sensitivity or resistance [204].

Since aPDT is still under development, optimal models are necessary to investigate not only the effectiveness of treatment after microbial infections, the response of the immune system, PS cytotoxicity, but also the penetration depth of the light beam. *G. mellonella* seems to be an appropriate model for the study of different aspects of aPDT during infections caused by bacterial and fungal—mono- but also dual or multi-species biofilms [18,58,209,210,211,212]. Moreover, PDI can be tested in combination with other bioactive molecules, including antimicrobial drugs [93,213].

Recently, scientists began to test the effectiveness of aPDT on *G. mellonella* infected with *C. albicans* using different PSs, such as methylene blue [214], erythrosine, curcumin, or toluidine blue [121,209,213]. In the dissertation of Dr. Dadi, the PS phloxine B was tested for toxicity in *Galleria* larvae, and even a 0.5 mM concentration did not exhibit any effect on *G. mellonella* survival [215].

**Figure 1:**
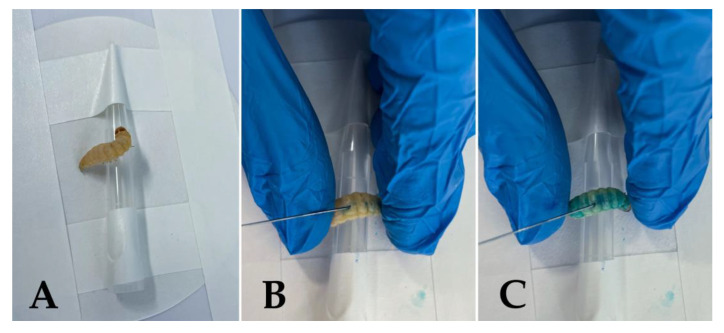
Inoculation of *G. mellonella* larva with 1 mM methylene blue in PBS. (**A**) An injection station for the simple manipulation of the larva during inoculation, which involves taping a filter paper disc to the table and a 1000 µL-disposal tip onto the filter paper. (**B**) Injection of the larva: the *G. mellonella* larva is gently held over the tip using the fingers, or tweezers, with the prolegs at the tail end of the larva’s body visible. The needle is carefully inserted into a proleg, angling the needle toward the head of the larva, and 10 µL of methylene blue in PBS is administrated. A different proleg should be used for PS administration than for the previous inoculation of the pathogen to avoid contamination. (**C**) During the release of the methylene blue, the larva visibly turns blue.

The protocol for simple testing is as follows. After the inoculation of the larvae with a cell suspension of a known density (this should usually be estimated in a preliminary experiment for each microbial genus or species), the tested PS, diluted in sterile phosphate buffer saline (PBS) to the desired concentration, is applied to the *G*. *mellonella* larvae, usually by the inoculation method. Using a 10 µL Hamilton syringe, 10 µL aliquots of the cell suspension are administrated into the hemocoel of each caterpillar via the proleg at the tail end of the larva’s body, followed by the administration of the PS via the opposite proleg (Figure 1) [58,209,210,212,216,217]. For some purposes, the PS can also be applied locally, as described in a study by Figueiredo-Godoi *et al.* (2022) [18], who used *G. mellonella* for a burn model infected with *A. baumannii*.

**Figure 2:**
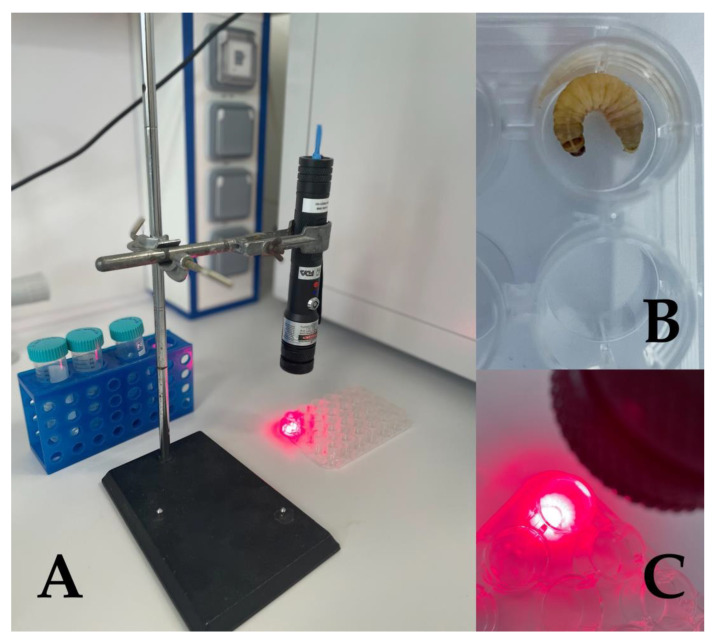
The process of the irradiation of *G. mellonella* larva with a red laser. (**A**) PDT assembly; (**B**) the *G. mellonella* larva was positioned in the well of a 24-well microtiter plate; (**C**) irradiation of the larva´s body with the red laser (λ = 660 nm, 190 mW/cm^2^). The distance between the larva and the laser was 10 cm and the duration of irradiation was 2 min, which corresponded to an energy delivery of 23 J.

The application of the PS is followed by irradiation with light of an appropriate wavelength, and the delivered energy is calculated, taking into account the duration of the irradiation, to determine the total effectivity of irradiation—fluence. The PS application should be approximately 10–30 min before the irradiation, allowing the PS to penetrate the tissue and finally the microorganisms. During irradiation, energy transfer from the PS in the presence of oxygen results in the generation of ROS. One molecule of PS can activate many atoms of activated oxygen. However, it should be considered that the diffusion of the activated oxygen is limited. Another limitation is the proximity to the PS, as objects distant from it may be subjected to limited or no damage [209,213,218]. The irradiation of *G. mellonella* is also a critical step, as it is important to ensure the proper delivery of the light to cover the desired area of the insect body completely. For this purpose, the larva should be maintained in a 24-well microtiter plate throughout the irradiation process (Figure 2). To prevent the organism from moving around, it is advisable to perform the irradiation of each larva separately, one by one, and to keep the larva inside its well using forceps. After performing aPDT, the larvae are incubated in Petri dishes at the required temperature (usually at 37 °C in the dark). Every experiment must include a control group of *G. mellonella* larvae that do not receive any injections to monitor the overall quality of the larvae over the course of the experiment, as well as a PBS injection control group to ensure that death was not due to trauma. The survival of aPDT-treated larvae is recorded daily or hourly, according to a pathological scoring system proposed by Loh *et al.* (2013) [78] taking into account a few attributes, such as movement activity, melanization, or cocoon formation. Figure 2 illustrates the irradiation of *G. mellonella* larvae with a red laser, and how this is processed in the laboratory of Prof. Bujdáková *et al*.

The key factor in PDT (aPDI) effectiveness is PS, which must meet the compatibility parameters and have high efficiency. Absorption in the red and near-infrared spectrum is also advantageous, as red light is relatively favorable to the treated host. The PS needs to exhibit only local toxicity, even after light activation. A high level of ROS yield is also assumed during irradiation [219,220,221].

**Figure 3:**
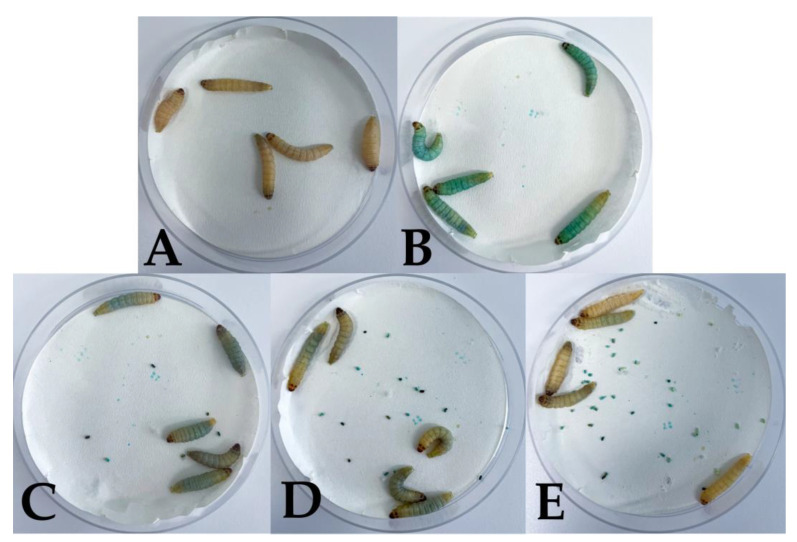
Testing the toxicity of 1 mM methylene blue on *G. mellonella* larvae. (**A**) Larvae without an injection—control group. (**B**) *G. mellonella* inoculated with 1 mM methylene blue in PBS. Immediately after inoculation, the larvae were visibly colored blue. (**C**) *G. mellonella* after 24 h. The larvae slowly began to discolor, and their excrement was blue. (**D**) *G. mellonella* after 48 h. The discoloration was progressing. (**E**) *G. mellonella* after 120 h. The larvae were completely discolored and resembled the control group without any harmful effects.

Phenothiazinium dyes are the most common PSs used in PDT performed on the *G. mellonella* model [58,210,212,216,222]. During the administration of the desired PS into the larva´s hemocoel, the body of the larva becomes colored, which is an accompanying phenomenon. The intensity of the color depends on the concentration of the PS used. Over the course of the experiment, the larvae excrete the dye and become discolored (Figure 3).

*G. mellonella* larvae have been found to be versatile in several studies that evaluated a PS used in aPDT [210,211,223]. De França *et* *al.* (2021) tested *in vitro* the anti-tumor effect and skin permeation/retention of a green fluorescence pyrene-based dye for aPDT, and they used the *G. mellonella* model to determine PS toxicity [224]. Rigotto Caruso *et al.* (2021) evaluated the antifungal activity of aPDT *in vitro* with different phenothiazinium PSs (methylene blue, new methylene blue N, and new methylene blue N Zinc) in combination with biosynthesized silver nanoparticles. The toxicity of all the tested compounds during their study was verified in the *G. mellonella* model [225]. The tests performed in a study by Malacarne *et al.* (2023) evaluated the toxicity of porphyrin PS on *G. mellonella* larvae and its cytotoxicity on hemocytes. No dark toxicity of PS was observed, even at the highest concentrations, and even with the longest incubation period (72 h). The intracellular localization of porphyrin PS was assessed using fluorescence microscopy after the hemocytes were isolated and collected from the hemolymph of inoculated larvae [226].

Nowadays, a relatively wide range of PSs are available, including phenothiazine dyes, porphyrins, chlorines, and phthalocyanines. In addition to synthetic ones, natural substances such as chlorophyllin, curcumin, and hypericin have also been studied [18,209,217,227,228,229].

The development of optimal light sources for PS is important for effective aPDT. Many PSs used for *in vivo* testing are activated by a red light with a wavelength between 630 and 700 nm. The source of light is a light-emitting diode (LED light) or diode laser. The irradiation itself must not affect the survival of the larvae [18,58,212,222,223,230,231].

During the interaction of the tissue with a light beam, most of the light is absorbed, scattered, or transmitted, and only 4–7% is reflected. Pigmented tissue areas absorb light preferentially compared to less pigmented ones [231]. aPDT can also be enhanced by increasing the PS concentration. However, higher concentrations of PS can result in the formation of aggregates, leading to an optical shielding phenomenon that can reduce the killing of microbial cells [232,233].

Merigo *et al.* (2017) studied the use of different laser energy densities (650 nm, 450 nm, and 532 nm) with or without different types of PSs (toluidine blue, curcumin, and erythrosine) in *C. albicans* infections. The authors *suggested that* laser irradiation in combination with an appropriate PS, and even the use of laser irradiation alone, were shown to be effective at controlling candidiasis using the *G. mellonella* model [209].

In a study by Figueiredo-Godoi *et al*. (2019), red laser penetration, delivered at different fluencies (660 nm, 6 and 15 J/cm^2^), and the distribution of light in the tissue of *G. mellonella* larvae was investigated using a power meter and CCD camera. The images were analyzed according to the interactive 3D Surface Plot plugin of the Image J program. Subsequently, the concentration of the PS—methylene blue (100 µM) which allowed the best light distribution over the thickness of the larvae’s body after administration was chosen for the aPDT assays. The authors observed that without the PS, the beginning of the light distribution in the cuticle occurred at 0.36 mm, and remained for up to 2.5 mm. In association with 100 µM methylene blue, the light distribution occurred at 0.27 mm and extended up to 2.45 mm below the cuticle. These findings suggested that laser irradiation in association with the proper PS can enhance light distribution in the cuticle [58].

Bispo *et al.* (2020) performed bacteria-targeted aPDT, which relied on the combination of a bacteria-specific targeting agent and the light-induced generation of ROS by an appropriate PS in *G. mellonella*. They conjugated the near-infrared PS IRDye700DX to a fully human monoclonal antibody, specific to the immunodominant staphylococcal antigen A (IsaA), creating a novel photo-immunoconjugate. They proved that aPDT with 1D9-700DX was highly effective at treating *G*. *mellonella* infected with a methicillin-resistant strain. Despite the observed relapse in the bacterial burden 48 h after aPDT, this relapse was not lethal to the larvae, as there were increased survival rates (~80%) 72 h after treatment. The authors suggested that the increased survival could be attributed to the innate larval immune defenses. The authors concluded that aPDT with 1D9-700DX reduced the bacterial burden to such an extent that the host’s immune responses could overcome infections caused by multidrug-resistant *S*. *aureus* [234].

Chibebe *et al.* (2013) used *G. mellonella* for testing the effectiveness of aPDT in the presence of methylene blue. They demonstrated the prolonged survival of *G. mellonella* after infection with *C. albicans*. The fungal burden of *G. mellonella* hemolymph was reduced, and the administration of fluconazole—either before or after exposing the larvae, infected with fluconazole-resistant *C. albicans*, to aPDT—significantly prolonged their survival compared to the control group. These findings suggested that aPDT combined with conventional antimicrobial drugs could have a synergistic effect, representing an effective strategy for the treatment of infections caused by resistant clinical strains [213].

The *G. mellonella* model has been used to identify the regulation of innate immunity by aPDT [93,210,216,223]. Dos Santos *et al.* (2017) reported that aPDT activated the *G. mellonella* immune system by increasing the circulation of hemocytes against *Porphyromonas gingivalis* infection and by attenuating infection, prolonging the survival of the infected group of larvae [216]. A study by Huang *et al.* (2020) [223] confirmed that aPDT had immunomodulatory effects; they demonstrated that 5-aminolevulinic acid (ALA)-mediated aPDT increased hemocyte density. Moreover, the extracted hemocytes after ALA-mediated aPDT had increased susceptibility to *C. albicans* and *S. aureus*.

Paziani *et al.* (2019) found that the total hemocyte count after aPDT with phenothiazinium PSs (methylene blue, new methylene blue, and pentacyclic phenothiazinium photosensitizer S137) of infected *G. mellonella* increased in larvae hemolymph, whereas the fungal burden was decreased. The increase in the cellular immune response was correlated to the increase in larval survival and decrease in fungal burden. The survival levels of infected larvae with *Fusarium keratoplasticum* were 70, 60, and 80% after aPDT with methylene blue (1500 μM), new methylene blue (200 μM), and S137 (200 μM), respectively, 10 days after infection. The survival levels of larvae infected with *Fusarium moniliforme* were 40, 10, and 100% after aPDT with methylene blue (1500 μM), new methylene blue (200 μM), and S137 (200 μM), respectively, 10 days after infection. Thus, the larvae infected with *F. keratoplasticum and F. moniliforme, which were found to be resistant to itraconazole and posaconazole,* survived because the cellular immune system response of *G. mellonella* acted effectively [210].

Table 1 summarizes a list of published works studying the effectiveness of aPDT or PS toxicity on *G. mellonella* using various conditions of aPDT, tested PSs, and microorganisms selected for infection.

## 4. Conclusions

The information summarized in this review points to the versatile use of *G. mellonella* in biological research. This model has also been proven to be highly suitable for the study of aPDT, despite some limitations, for example, the availability of oxygen in the tissues or the delivery of light into the tissue, while achieving high efficiency in terms of irradiation. Of course, the biocompatibility and photoactivity of the PS are the necessary conditions for the overall effectiveness of aPDT. Many available and generally known techniques can be adopted with *G. mellonella* in terms of the experiment design and expected results, but the protocols must be optimized, taking into consideration the specificity of this model organism. It is also necessary to think about the fact that the *G. mellonella* larvae must meet the basic standard conditions for breeding and preservation to avoid discrepancies in the obtained results. In summary, *G. mellonella* has great potential for experimental studies of aPDT.

## Figures and Tables

**Table 1:** List of published works focused on testing PS toxicity or aPDT on microbial infection using *G. mellonella* larvae as a model.

Photosensitizer	Light Source	Energy	Microorganism	Authors	Reference
Methylene blue 0.2 mg/mL	660 nm red light device composed of 48 LEDs	30 J/cm^2^	*Acinetobacter baumannii*	Figueiredo-Godoi *et al.* (2022)	[18]
Fotenticine 1.2 mg/mL	660 nm red light device composed of 48 LEDs	30 J/cm^2^	*Acinetobacter baumannii*	Figueiredo-Godoi *et al.* (2022)	[18]
Methylene blue 75–600 μM	660 nm red laser light	6 J/cm^2^ and 15 J/cm^2^	*C. albicans*	Figueiredo-Godoi *et al*. (2019)	[58]
Methylene blue 1 mM	660 ± 15 nm broadband non-coherent red light source	0.45–18 J/cm^2^	*Enterococcus faecium*	Chibebe Junior *et al.* (2013)	[93]
Erythrosine 100 μM	532 nm green diode laser	10 J/cm^2^	*C. albicans*	Merigo *et al.* (2017)	[209]
Curcumin 100 μM	405 nm blue-violet diode laser	10 J/cm^2^	*C. albicans*	Merigo *et al.* (2017)	[209]
Toluidine blue 10 μM	650 nm red diode laser	10 J/cm^2^	*C. albicans*	Merigo *et al.* (2017)	[209]
Methylene blue 750–3000 μM	An array of 96 light-emitting diodes with an emission peak at 635 nm and integrated irradiance from 570 to 670 nm	15 J/cm^2^	*Fusarium keratoplasticum, F. moniliforme*	Paziani *et al.* (2019)	[210]
New methylene blue N 100–400 μM	An array of 96 light-emitting diodes with an emission peak at 635 nm and integrated irradiance from 570 to 670 nm	15 J/cm^2^	*Fusarium keratoplasticum, F. moniliforme*	Paziani *et al.* (2019)	[210]
Pentacyclic phenothiazinium photosensitizer S137 100–400 μM	An array of 96 light-emitting diodes with an emission peak at 635 nm and integrated irradiance from 570 to 670 nm	15 J/cm^2^	*Fusarium keratoplasticum, F. moniliforme*	Paziani *et al.* (2019)	[210]
Curcumin 50 μg/mL	440–480 nm LED source	1.2 J/cm^2^	*Streptococcus mutants*	Sanches *et al.* (2019)	[211]
Diacetylcurcumin 50 μg/mL	440–480 nm LED source	1.2 J/cm^2^	*Streptococcus mutants*	Sanches *et al.* (2019)	[211]
Methylene blue 100 μM	660 nm LED source	3–18 J/cm^2^	*Escherichia coli*	Garcez *et al.* (2020)	[212]
Methylene blue 1 mM	660 ± 15 nm broadband non-coherent red light source	0.45–18 J/cm^2^	*C. albicans*	Chibebe Junior *et al.* (2013)	[213]
Methylene blue 600 mM	660 nm red laser light	15 J/cm^2^	*Porphyromonas gingivalis*	Dos Santos *et al.* (2017)	[216]
Curcuma longa L. Extract 100 mg/mL	---	---	*---*	Marques Meccatti *et al.* (2022)	[217]
Curcumin 200 μg/mL	---	---	*---*	Marques Meccatti *et al.* (2022)	[217]
Methylene blue Concentration not specified	An array of 96 light-emitting diodes with an emission peak at 635 nm	15 J/cm^2^	*C. albicans, C. auris*	Grizante Barião *et al.* (2022)	[222]
New methylene blue N Concentration not specified	An array of 96 light-emitting diodes with an emission peak at 635 nm	15 J/cm^2^	*C. albicans, C. auris*	Grizante Barião *et al.* (2022)	[222]
Toluidine blue O Concentration not specified	An array of 96 light-emitting diodes with an emission peak at 635 nm	15 J/cm^2^	*C. albicans, C. auris*	Grizante Barião *et al.* (2022)	[222]
Pentacyclic phenothiazinium photosensitizer S137 Concentration not specified	An array of 96 light-emitting diodes with an emission peak at 635 nm	15 J/cm^2^	*C. albicans, C. auris*	Grizante Barião *et al.* (2022)	[222]
Methylene blue 10–500 mM	630 nm red light-emitting diode device	Not specified	*Fonsecaea monophora*	Huang *et al.* (2020)	[223]
5-aminolevulinic acid 10–500 mM	630 nm red light-emitting diode device	Not specified	*Fonsecaea monophora*	Huang *et al.* (2020)	[223]
